# Evidences for lipid involvement in SARS-CoV-2 cytopathogenesis

**DOI:** 10.1038/s41419-021-03527-9

**Published:** 2021-03-12

**Authors:** Roberta Nardacci, Francesca Colavita, Concetta Castilletti, Daniele Lapa, Giulia Matusali, Silvia Meschi, Franca Del Nonno, Daniele Colombo, Maria Rosaria Capobianchi, Alimuddin Zumla, Giuseppe Ippolito, Mauro Piacentini, Laura Falasca

**Affiliations:** 1 Laboratory of Electron Microscopy, National Institute for Infectious Diseases “Lazzaro Spallanzani”, IRCCS, Rome, Italy; 2 Laboratory of Virology, National Institute for Infectious Diseases “Lazzaro Spallanzani”, IRCCS, Rome, Italy; 3 Pathology Unit, National Institute for Infectious Diseases “Lazzaro Spallanzani”, IRCCS, Rome, Italy; 4 Department of Infection, Division of Infection and Immunity, University College London and NIHR Biomedical Research Centre, UCL Hospitals NHS Foundation Trust, London, UK; 5 Scientific Direction; National Institute for Infectious Diseases “Lazzaro Spallanzani”, IRCCS, Rome, Italy; 6 Department of Biology, University of Rome “Tor Vergata”, Rome, Italy

**Keywords:** Viral infection, Infection

## Abstract

The pathogenesis of SARS-CoV-2 remains to be completely understood, and detailed SARS-CoV-2 cellular cytopathic effects requires definition. We performed a comparative ultrastructural study of SARS-CoV-1 and SARS-CoV-2 infection in Vero E6 cells and in lungs from deceased COVID-19 patients. SARS-CoV-2 induces rapid death associated with profound ultrastructural changes in Vero cells. Type II pneumocytes in lung tissue showed prominent altered features with numerous vacuoles and swollen mitochondria with presence of abundant lipid droplets. The accumulation of lipids was the most striking finding we observed in SARS-CoV-2 infected cells, both in vitro and in the lungs of patients, suggesting that lipids can be involved in SARS-CoV-2 pathogenesis. Considering that in most cases, COVID-19 patients show alteration of blood cholesterol and lipoprotein homeostasis, our findings highlight a peculiar important topic that can suggest new approaches for pharmacological treatment to contrast the pathogenicity of SARS-CoV-2.

## Introduction

Since the first discovery of SARS-CoV-2, as a novel human zoonotic pathogen in late December 2019 (ref. ^1^), there have been 1.5 millions of deaths from COVID-19 disease reported by the WHO as of December 2020 (ref. ^2^). Current knowledge of COVID-19 pathogenesis is evolving and various pathogenetic mechanisms for COVID-19 have been proposed, including direct cytopathic effects, ischemic injury, and excessive aberrant immune responses^3–11^. SARS-CoV-2 infects the host cells using the angiotensin converting enzyme 2 (ACE2) receptor^12^, which is expressed in cells and vessels of several organs, including the lung, heart, kidney, and intestine, but ultrastructural studies carried out so far show rather discordant findings concerning the presence of viral particles inside different tissues^13–15^. The definition of the SARS-CoV-2 intracellular localization and its cytopathic effects are important for elucidating the pathogenetic mechanisms of SARS-CoV-2.

Cholesterol has recently recognized to be involved in the SARS-CoV-2 entry into the host cell^16^, however lipid rearrangements in SARS-CoV-2 host cells have not been explored so far.

In vitro cytopathic studies of SARS-CoV-2 using cell lines may not capture the in vivo pathology and thus performing studies in parallel is important. Based on this assumption we performed a comparative ultrastructural study of SARS-CoV-2 infection in Vero E6 cells and lung tissue from patients who died of COVID-19 disease. We also investigated the effects of SARS-CoV-2 on Vero cells, compared to effects of SARS-CoV-1.

## Results

### SARS-CoV-2 morphology

**Fig. 1:**
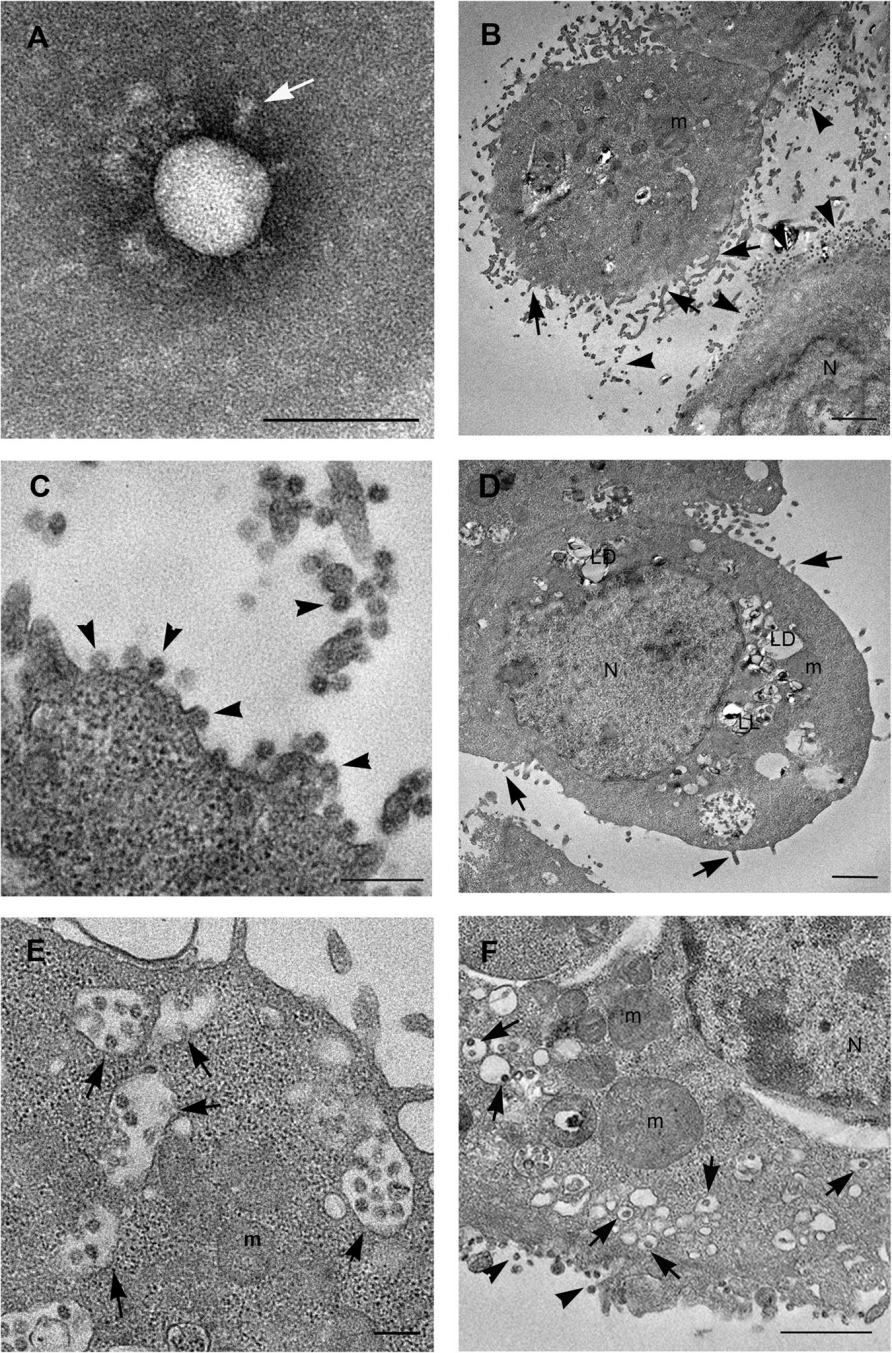
Electron microscopy images of SARS-CoV-2 virus and infected Vero cells. **A** Negative staining electron microscopy micrographs of SARS-CoV-2 particle. The virion display a spherical shape, on the surface cone-like shaped spikes are visible (white arrow). **B**–**D** Infected cells show numerous viruses associated to the plasma membrane (arrowheads) especially found along microvillous projections (arrows). Many lipid droplets (LD) and lipolysosomes (LL) are visible (**D**). **E** A great number of vacuoles are present in the cell cytoplasm, many of which contain viral particles (arrows). **F** Other vesicles, small in size, contain single viral particles, resembled the “spherules” described for other coronaviruses (arrows). Viruses are visible along the plasma membrane (arrowheads). Numerous free ribosomes are diffused in the cell cytosol. N nucleus, m mitochondria, LD lipid droplets, LL lipolysosomes. Scale bars: **A** = 100 nm; **B**, **D**, **F** = 1 μm; **C**, **E** = 200 nm.

The morphology of SARS-CoV-2 was characterized by negative staining electron microscopy. Purified viral particles revealed a spherical (Fig. 1A) or slightly pleomorphic shapes (Fig. S1A, C, D). On the surface of the virions the typical rim of cone-shaped spikes was identified, but their distribution was not as regular as usually reported for other Coronavirus, in fact, they appeared in multi-aggregated fashion (Figs. 1A and S1). The diameter of the viruses ranged from 80 to 102 nm (average size 93.61 nm), while the length of the spikes ranged from 9 to 12.5 nm (average length 10.99 nm). Some of the viral particles showed part of the ribonucleic-protein material extruding from rupture of the envelope (Fig. S1C).

### Cytophatic effects caused by SARS-CoV-2 and SARS-CoV-1 infection

SARS-CoV-2-infected Vero E6 cells started to show evident cytopathic effects from 24 h.p.i. (Fig. S2A), progressing toward rounding and detaching of the cells at 48 h.p.i (Fig. S2B). SARS-CoV-1 infected cells displayed similar onset and extent of cytopathic effects (Fig. S2C, D). Light microscopy of thin-sections from resin embedded samples, showed that at 24 h post infection with SARS-CoV-2, many cells lose the typical elongated shape of uninfected cells (Fig. S3A) and become roundish and rich in plasma membrane extroflessions (Fig. S3B). After 48 h from the infection cell morphology further changed dramatically. Most cells appeared swollen and showed numerous cytoplasmatic vacuoles; in contrast, other cells appeared dark suggesting that cell shrinkage occurred (Fig. S3C, D).

### Electron microscopy analysis of SARS-CoV-2-infected Vero cells

**Fig. 2:**
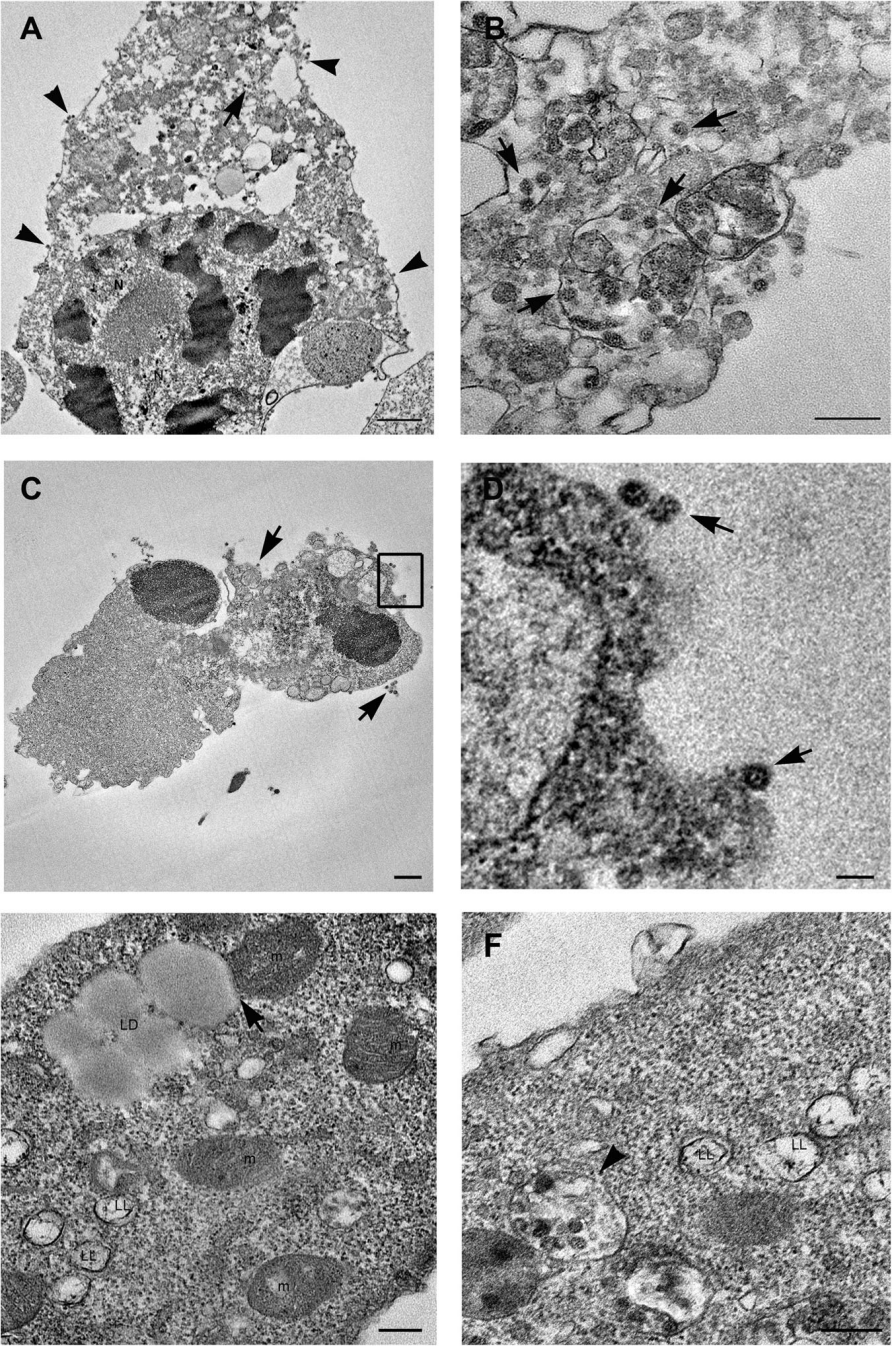
Electron microscopy features of SARS-CoV-2-infected dying cells. **A**, **B** A dying cell shows an advanced stage of degeneration. The nucleus shows condensed areas and the cytoplasm results empty due to the presence of a high number vacuoles, some of which containing viral particles (arrows). Numerous viral particles are also visible at the cell membrane (arrowheads). **C** Cell remnants showing viral particles outside of cell (arrows). **D** Higher magnification of the boxed area visible in **C** shows viral particles (arrows). **E** Lipid droplets (LD) are present inside infected cells, some of which are in contact with mitochondria (arrow). Lipolysosomes (LL) with external membrane and whorls are detected. Mitochondria (m) show swollen cristae. **F** Viral particles in structures resembling lipolysosomes (LL) are visible (arrowhead). N nucleus, m mitochondria, LD lipid droplets, LL lipolysosomes. Scale bars: **A**, **C** = 1 um; **D** = 100 nm; **B**, **E**, **F** = 200 nm.

Transmission electron microscopy analysis of SARS-CoV-2-infected Vero cells, at 24 h post infection, showed several round shaped cells, with prominent presence of filopodia at the plasma membrane (Fig. 1B). Many mature viral particles were visible at the cell surface (Fig. 1B, C). Inside the cells, SARS-CoV-2 particles were detected in virus containing compartments (VCC) that were with different size and shape (Fig. 1E, F). Group of virions were enclosed in single membrane vacuoles, similar to endosomes (Fig. 1E). Other vesicles, small in size, containing single viral particles, resembled the “spherules” described for other coronaviruses (Fig. 1F)^17,18^. At 48 h post infection most cells showed strong signs of degeneration and many were clearly dying (Fig. 2). Some cells showed extensive vacuolization of the cytoplasm and depletion of all organelles (Fig. 2A). Vacuoles containing viruses were still detectable in necrotic cells (Fig. 2B). Some cells seemed to die with morphological features of both apoptosis and necrosis, in which condensed cellular contents was dispersed by means of plasma membrane leakage. Free released viruses were also observed associated with cell remnants (Fig. 2C, D). The most striking finding we observed in infected cells was the presence of numerous lipid droplets (LDs) significantly increasing with time of infection (Fig. S5A). LDs showed variable size and morphology (Fig. 2E, F). Some of them displayed homogeneous content, the typical feature of lipid storage without encompassing membrane (Fig. 2E). Other droplets presented an external dark membrane (Fig. 2E, F) and were identified as lipolysomes described in humans with abnormalities in lipid metabolism^19^. Interestingly, mitochondria in contact with the lipid droplets were often found (Fig. 2E). Quantification of the number of contacts per cell showed a trend to an increased value at 48 h post infection compared to 24 h post infection (Fig. S5B).

In most cells mitochondria appeared altered and display swollen cristae (Fig. 2F). Of note, virus particles were also found associated with lipolysosomes suggesting that they can play an important step in virus assembly (Fig. 2F).

### Electron microscopy analysis of SARS-CoV-1-infected Vero cells

**Fig. 3:**
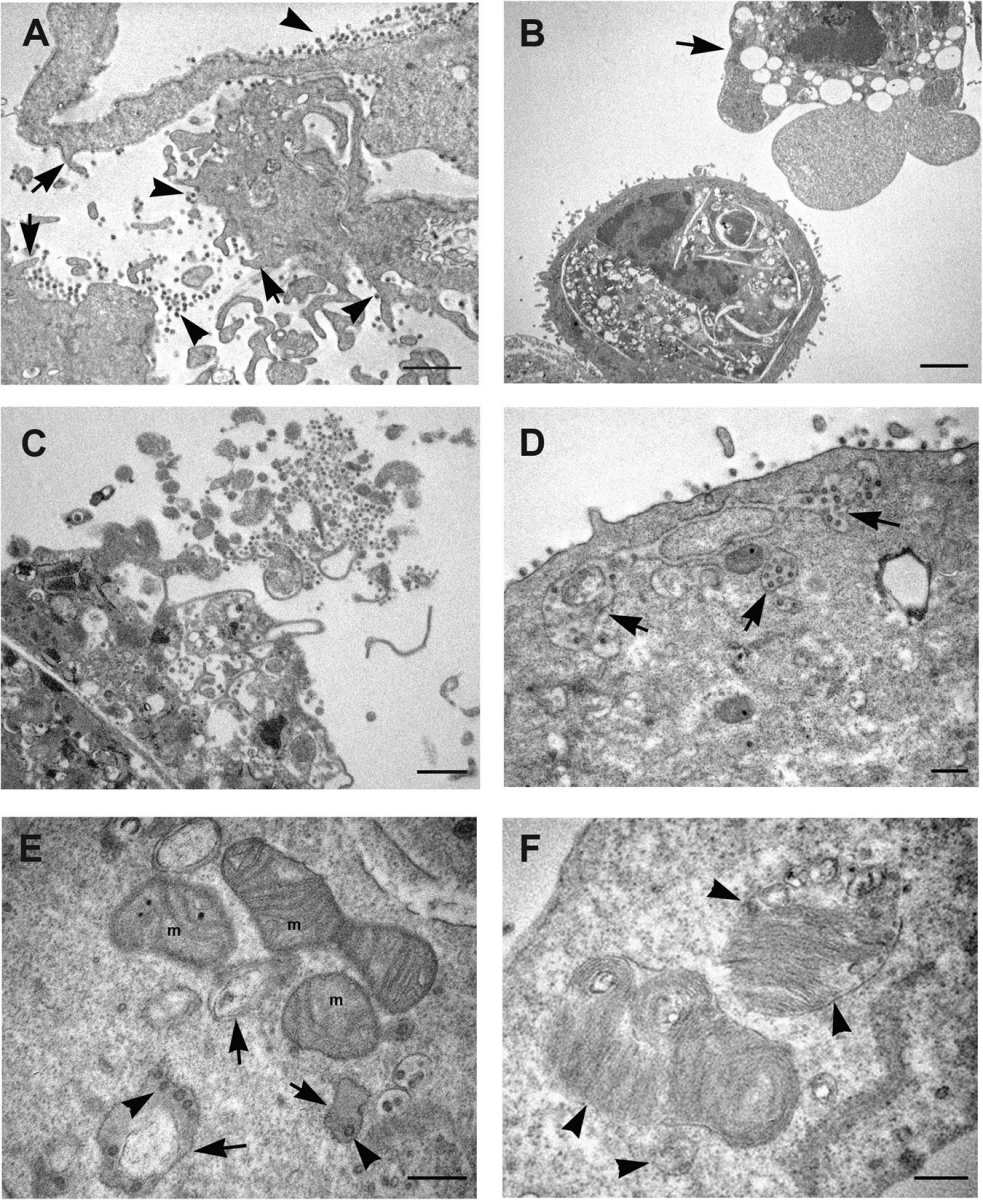
Electron microscopy micrographs of SARS-CoV-1 infected Vero cells. **A** Infected cells show a rough aspect, and many microvillous projections are visible all over the cell surface (arrows). Numerous virus particles are visible at the cell membrane or in the extracellular space (arrowheads). **B** Cytopathic effect of viral infection generates in a number of cells condensation of cytoplasm and apoptotic cell death (arrow). **C** Cellular lysis with release of cell material with associated virus particles is observed. **D** Groups of viral particles were found inside vesicles near the plasma membrane of infected cells (arrows). **E** Morphological modifications of mitochondria (m) are shown, consisting in swelling with a reduction in membrane cristae amount, leading to the formation of vesicles (arrows). Virus particles are visible inside these vesicles (arrowheads). **F** SARS-CoV-1 infected cells display the presence of multilamellar structure: at the periphery of the structure ribosome-carrying membrane are visible (arrowheads). N nucleus, m mitochondria. Scale bars: **A**–**C** = 1 μm; **D**–**F** = 200 nm.

During the first 24 h post SARS-CoV-1 infection, Vero cells also showed modification of plasma membrane, which became enriched in filopodia and extroflessions associated with the presence of numerous virus particles (Fig. 3A). At 48 h post-infection appearance of vacuoles and roundish of cells was displayed. Some infected cells showed the formation of large septa, resulting in a more dramatic compartmentalization of cytoplasm compared to SARS-CoV-2 (Fig. 3B). The cytopathic effects induced by the SARS-CoV-1 infected cells resulted in both apoptotic (Fig. 3B) and necrotic cell death (Fig. 3C). Large vacuoles containing virus particles, resembling dilated spaces of endoplasmic reticulum were also detected (Fig. 3D). Mitochondria displayed loss of their typical morphology, they appeared swollen with progressive cristae disappearance, resulting in the formation of vesicles, which occasionally still maintain mitochondrial matrix. Virus particles were observed inside these vesicles, lining the membrane or in the process of pinching off (Fig. 3E). Virus particles were also found in deep association with particular multilamellar structures (Fig. 3F). In cells infected with SARS-CoV-1 we did not observe the presence of lipid droplets at any time of infection.

### SARS-CoV-2 infection and lipid droplets

**Fig. 4:**
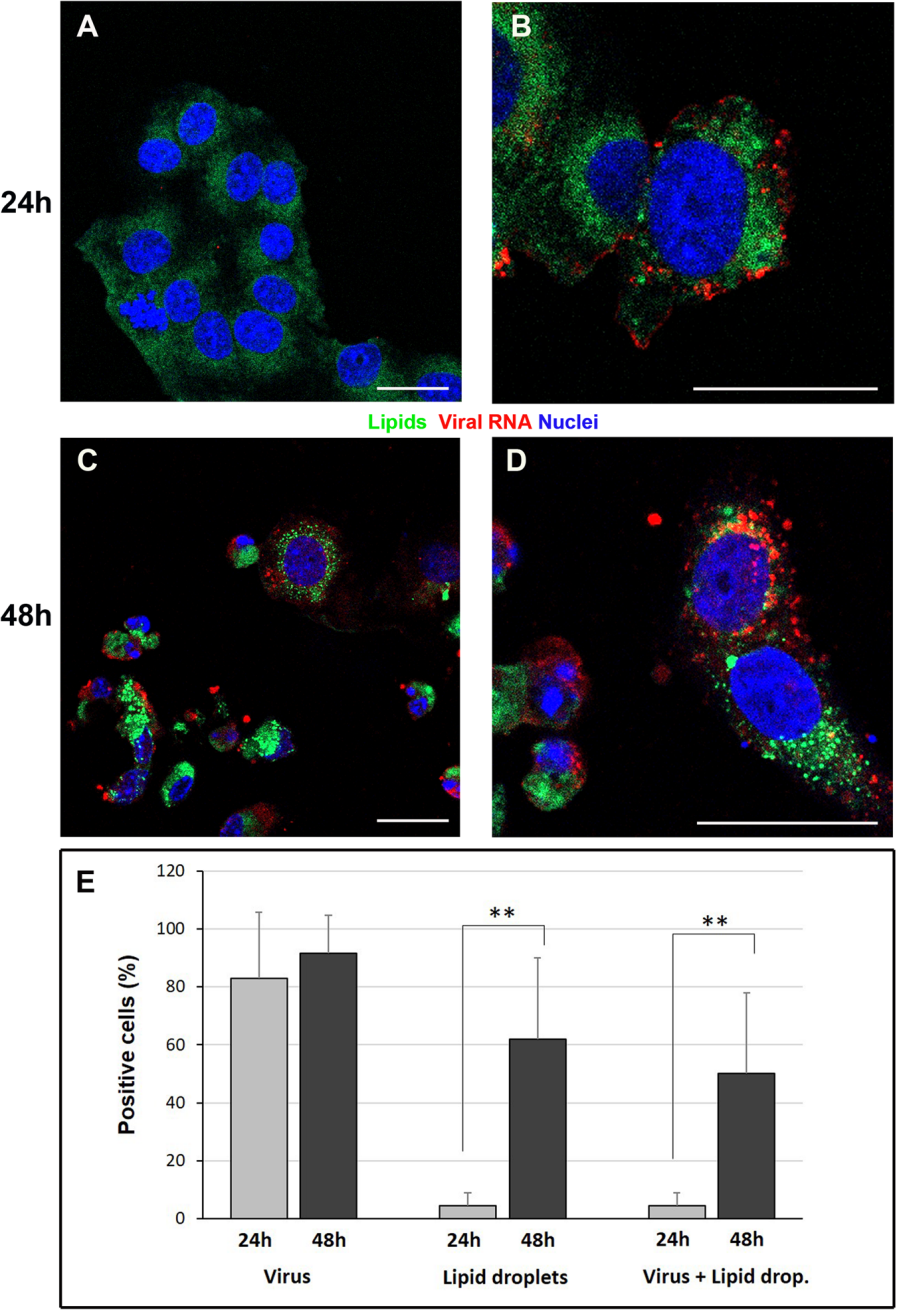
Confocal microscopy of SARS-CoV-2-infected Vero cells. Immunofluorescence dual-labeling detection of viral dsRNA and lipid droplets. **A** Control cells, display absence of dsRNA signal and diffuse staining with lipophilic dye. **B** At 24 h post infection some cells showing viral RNA (red) also present lipid droplets (green dots) in the cytoplasm. **C**, **D** Numerous lipid droplets are visible in the cell cytoplasms (green dots) of cells 48 h post infection. **E** The graph shows the percentage of cells displaying viral RNA, lipid droplets staining or both labeling at 24 h and 48 h post infection. Data are mean ± SD from at least three independent experiments, and each experiment included duplicate samples. Statistically significant difference is showed (**p < 0.01). dsRNA (red); lipid droplets (green). Nuclei are stained blue (DAPI). Scale bars: **A**–**D** = 5 μm.

To further analyze the presence of lipid droplets in SARS-CoV-2 infected cells, double staining was performed by indirect immunofluorescence for viral dsRNA and use of fluorescent lipophilic dye. dsRNA detection demonstrated that at 24 h p.i. the number of infected cells was more than 80% (Fig. 4E), and this number further increased at 48 h p.i. (Fig. 4E). The infected cells showed a significant increase in the number of cells presenting lipid droplets in their cytoplasm with time of infection (Fig. 4C–E). The presence of a diffuse dye staining in mock (controls) cells demonstrated the absence of lipid droplets (Fig. 4A).

### Histopathological examination of lung tissue from COVID-19 patients

**Fig. 5:**
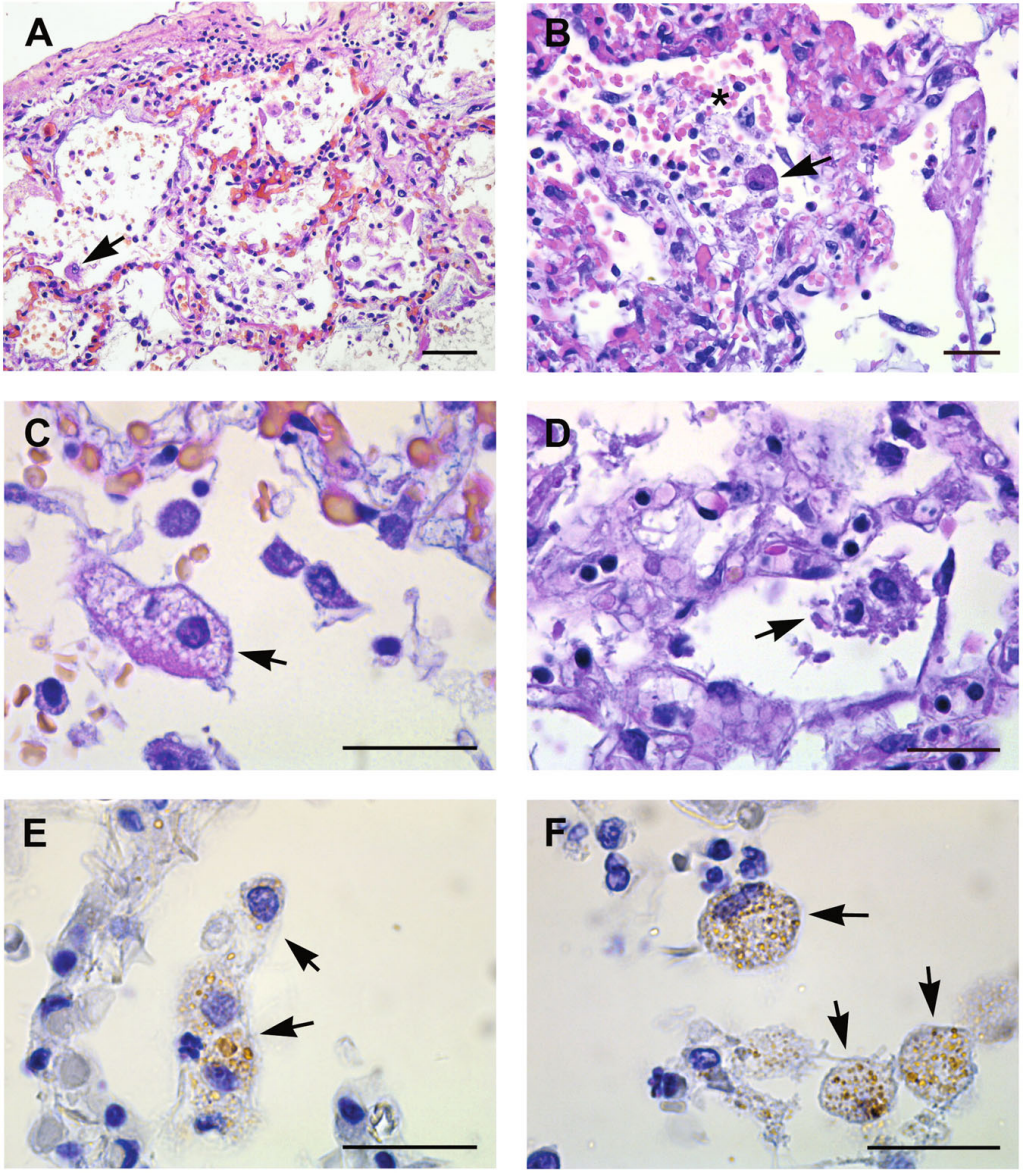
Histopathological changes of lung tissue from COVID-19 patients. **A**, **B** EE staining from lung tissue shows diffuse alveolar damage, with intra-alveolar inflammation, fibrin, and hyaline membranes. Hyperplasia of type II pneumocyte, characterized by amphophilic cytoplasm, large nuclei, and prominent nucleoli is visible (arrows). Necrotic cells are present in the alveolar space (asterisk) (**B**). **C** Type II pneumocyte showing signs of degeneration characterized by large nucleus, with fine and uniformly dispersed chromatin and cytoplasm vacuolization (arrow). **D** Degenerating pneumocytes showing tipical features of pyroptosis (arrow). **E**, **F** Immunohistochemistry anti-coronavirus revealed a focal distribution of the positivity (arrows). Scale bars: **A** = 14 μm; **B**–**F** = 7 μm.

Lung tissues samples obtained from 20 deceased COVID-19 patients were analyzed. Histopathological analysis performed on all cases, showed diffuse alveolar damage with hyaline membranes, fibrinous exudate, and inflammatory infiltrate (Fig. 5A, B). Necrotic cells and cell remnants were observed in the alveolar space (Fig. 5B). Damage of alveolar epithelium was associated with the presence of reactive type II pneumocyte, characterized by hyperplasia, amphophilic cytoplasm, large nuclei, and prominent nucleoli (Fig. 5B). Type II pneumocytes showed increased detachment from the alveolar walls and nuclear changes, making the nucleus difficult to distinguish (Fig. 4C, D). In addition, some pneumocytes displayed signs of degeneration such as highly vacuolated cytoplasm (Fig. 5C) or membranous blebs, suggestive of pyroptotic cell death (Fig. 5D). Anti-coronavirus immunolabeling revealed that the positivity was preferentially found in type II pneumocytes (Fig. 5E, F). Absence of viral staining was also found on endothelial cells (Fig. S4D).

### Electron microscopic examination of lung tissue from COVID-19 patients

**Fig. 6:**
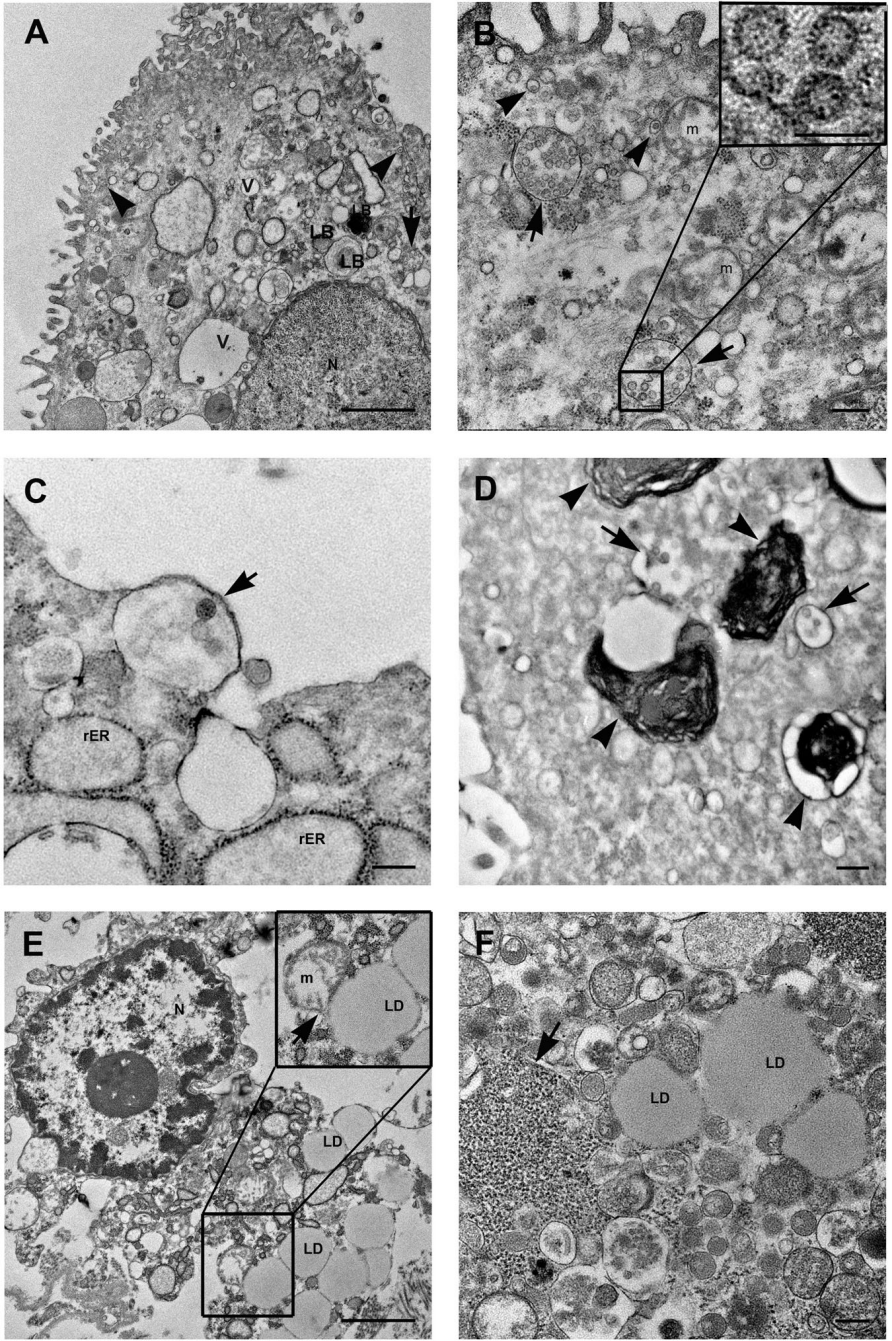
SARS-Cov-2 detecting on lung tissue from COVID-19 patients by transmission electron microscopy. **A**, **B** SARS-CoV-2 particles are detected in type II pneumocytes recognizable by the presence of lamellar bodies, containing surfactant (LB). Viruses are localized in virus containing compartments (arrows). Other vesicles, very small in size, contain single viral particles (arrowheads). Numerous vacuoles (V) are present in the cell cytoplasm. **B** Mitochondria (m) display swelling with a reduction in membrane cristae amount. Higher magnification of viral particles is visible in the boxed area: black dots are visible inside the viral particles due to cross section through the nucleocapside. **C** A vesicle containing viral particles (arrow) and enlargement of rough endoplasmic reticulum (rER) are shown. **D** Lamellar bodies, containing surfactant are visible (arrowheads). Arrows point to virus containing compartments. **E**, **F** Abundant lipid droplets (LD) are present inside lung cells. Mitochondria and LDs contacts sites were shown (arrow in insert panel). Free ribosomes are present in the cell cytosol, many of which are compartimentalized (arrow). N nucleus, m mitochondria, rER rough endoplasmic reticulum, LD lipid droplets, LB lamellar bodies, V vacuole. Scale bars: **A**, **E** = 1 μm; **B**–**D**, **F** = 200 nm; boxed area in **B** = 100 nm.

Electron microscopy analysis was performed on lung specimens from 4 out of 20 patients which resulted positive for SARS-CoV-2 PCR test on lung tissue. We obtained similar findings in all the four patients. The presence of SARS-CoV-2 virus was observed inside type II pneumocytes (Fig. 6A). As found in cultured infected cells, virions were observed enclosed in single-layered cytoplasmic compartments of variable size, containing numerous viral particles, or as sole particles into the “Spherules” (Fig. 6A, B). The pneumocytes showed altered morphological features, for example the nucleus appeared with finely and uniformly dispersed chromatin (Fig. 6A) or with convoluted profile and marginated chromatin alternated to cleared regions (Fig. 6E). Those cells displayed organelles injury comparable with those observed in Vero cells infected with the SARS-CoV-2. The pneumocytes showed the presence of numerous vacuoles and swollen mitochondria (Fig. 6A, C). In the infected cells the rough endoplasmic reticulum and free ribosomes, (which are typically abundant in type II pneumocytes, due to the production of surfactant), were respectively enlarged and compartmentalized (Fig. 6C, F). Of note, in agreement to what observed in cultured cells, type II pneumocytes showed an unusual presence of lipid droplets (LDs) accumulation (Fig. 6E, F). Contacts sites between mitochondria and LDs were observed (Fig. 6E).

Lipid accumulation in lung tissue was also confirmed by fluorescent staining (Fig. S4). Quantification analysis showed a mean number of 5.24 ± 2.78 droplets per cell, with a range among different patients comprised between 2.9 and 8.3.

Dying pneumocytes had morphological features which did not resemble neither necrosis nor classical apoptosis. Some cells displayed condensation together with plasma membrane leakage and release of the cellular contents (Fig. 6E).

## Discussion

Three coronaviruses (CoVs) have crossed the species barrier to cause lethal zoonotic respiratory diseases in humans in the past 2 decades: Severe acute respiratory syndrome coronavirus (SARS-CoV) in 2003, Middle-East respiratory syndrome coronavirus (MERS-CoV), in 2012 and the SARS-CoV-2 in 2019^20,21^. Coronaviruses are positive-strand RNA viruses, that display a spherical morphology and spike glycoproteins projections on their surface which give them the typical crown-like shape under the electron microscope^22,23^. Since the mid-1960s, seven known human coronaviruses have been identified which involve the upper respiratory tract and the gastrointestinal tract, and generally cause mild diseases^24^. As other viruses, CoVs display an envelope that is formed by a lipid bilayer derived from the host cell membranes and for this reason intracellular membrane play a key role for coronaviruses replications. Coronavirus replication complexes, similar to other RNA viruses, appear to be anchored to membrane structures known as “viral factories”, derived from extensive modification of cell compartments^25^. These membranous structures not only harbor viral proteins but also contain a specific array of hijacked host factors, which collectively orchestrate a unique lipid micro-environment optimal for coronavirus replication^25–28^. Ultrastructural studies on Coronavirus genera have revealed that alpha- and beta-coronaviruses formed clusters of the double-membrane vesicle (DMV), sometimes linked by a convoluted membrane^29^, whereas the gamma-coronavirus IBV induced extensive paired membranes and smaller 60–80 nm spherules in addition to the DMVs^29–31^. In a recent study, it has been demonstrated that during human coronavirus infection the cell’s lipid profile is significantly altered^32,33^. A striking finding revealed by our study is the presence of numerous lipid droplets induced by SARS-CoV-2 infection, a major difference when compared to SARS-CoV-1 infection. Some (+)RNA viruses exploit lipid droplets (LDs) to acquire lipids for membrane or energy production to support their replication^34^. Lipid droplets found in SARS-Cov-2 infection, both in vitro and in type II pneumocytes, appear similar to those known to occur in hepatocytes as a consequence of HCV infection^35^. Lipid droplets with typical features of lipid storage, without encompassing membrane and translucent omogeneous appearance, were often observed. Other vesicles with the characteristics similar to lipolysosomes were also present, with an external membrane and whorls. In Vero E6 cells viral particles were also found associated with lipolysosomes suggesting that they can play a role in virus assembly. Another important observation, both in cultured cells and in lung, concerns mitochondria. A number of mitochondria were in close contact with lipids droplets. These contacts site have recognized as a key feature of lipid dynamics. The proximity of mitochondria and lipid droplets is necessary for the ATP production, via β-oxidation^36^. Besides, evidence for mitochondria and LDs contacts involvement in LD biogenesis has also been observed. In cells exposed to excess fatty acids this mechanism, increasing LD mass, protects against lipotoxic injury^37^. Recent in vitro findings described host lipid metabolic remodeling associated with coronaviruses propagation, suggesting that lipid metabolism regulation could be a common event for coronavirus infections^32^. Modulation of host lipid metabolism has been reported to be necessary for replication of virus, such as hepatitis C virus (HCV), and picornaviruses^35,38^. Several studies demonstrated that targeting host lipid metabolism by statins, allow to suppress viral replication of many positive-strand RNA viruses, such as Hepatitis C virus, Dengueviruses, Japaneseen-cephalitis virus, West Nile virus and influenza A virus. Statins, are able to destabilize lipid rafts involved in the viral replication phases, as they constitute packets of vesicles capable of concentrating virus replication factors^39,40^.

SARS-CoV-2 induced cell death is different to SARS-CoV-1 cytophatic effects^41,42^. Our results suggested that a distinct type of cell death, with morphological features of both apoptosis and necrosis, namely pyroptosis, could be induced by SARS-CoV-2^43^. COVID-19 is associated with a respiratory illness that may lead to severe pneumonia and acute respiratory distress syndrome (ARDS)^9^. Of note, in the pathogenesis of ARDS, pyroptosis may play an important role^44^.

In conclusion, our findings demonstrate peculiar ultrastructural changes induced by SARS-CoV-2 infection. In particular, our work revealed that SARS-CoV-2 infection induce the accumulation of lipid droplets, both in cultured cells and in type II pneumocytes of lung from infected patients. These findings highlight a novel important open topic which may indicate new targets to contrast the pathogenicity of SARS-CoV-2. Several studies demonstrated that targeting host lipid metabolism by statins allows to suppress viral replication of many positive-strand RNA viruses, such as Hepatitis C virus, Dengueviruses, West Nile virus, and influenza A virus. Our results suggest that clinical studies, to assess the efficacy of statins on COVID-19 patient, or interfering with key lipid metabolic pathway enzymes could represent yet unconsidered therapeutic perspective.

## Materials and methods

### SARS-CoV-2 and SARS-CoV-1 isolates

SARS-CoV-2: The first COVID-19 cases were identified on 31st January at our National Institute for Infectious Diseases IRCCS”Lazzaro Spallanzani”, Rome, Italy. SAR-CoV-2 was isolated and cultured from these patients^45^ and was used in this study.

SARS-CoV-1: SARS-CoV-1 used in this study was the Tor2 isolate kindly provided by National Microbiology Laboratory, Public Health Agency of Canada.

### VERO cell lines and infection with SARS-CoV-2 and SARS-CoV-1

Mammalian cell lines Vero E6 (ATCC® Number CRL-1586™) were cultured in Modified Eagle Medium (MEM, Sigma Aldrich) containing 10% fetal bovine serum (FBS), at 37 °C in a 5% CO2 atmosphere. Sub-confluent cells were exposed to SARS-CoV-2 INMI1 isolate (named 2019-nCoV/Italy-INMI1, GISAID accession number: EPI_ISL_410546) obtained from sputum sample from the first COVID-19 patient reported in Italy in January 2019 and hospitalized at INMI, for 1 h at 37 °C at a multiplicity of infection (MOI) of 0.01.

At the end of the adsorption period, cells were washed, and fresh medium was added. The same method was used for infection of Vero cells with SARS-CoV-1. Uninfected Vero cells were used as controls. Three or more biological replicates were harvested at each described time.

### Negative staining

Purified SARS-CoV-2 viral suspensions were fixed in 2.5% glutaraldehyde and allowed to adsorb onto a formvar carbon-coated grid for a few minutes before being stained with 1% phosphotungstic acid for 1 min. The excess fluid was blotted and the grid left to dry before viewing under a transmission electron microscope JEOL JEM 2100 Plus (Japan Electron Optics Laboratory Co. Ltd. Tokyo). Images were captured digitally with a digital camera TVIPS (Tietz Video and Image Processing Systems GmbH. Gauting, Germany).

### Lung tissues

Lung tissue samples were obtained from post-mortem examination of 20 consecutive SARS-CoV-2-infected patients, performed at the National Institute for Infectious Diseases Lazzaro Spallanzani-IRCCS Hospital (Rome, Italy). All patients were diagnosed as COVID-19 by PCR test for SARS-CoV-2 (using RealStar® SARS-CoV-2 RT-PCR Kit 1.0 (Altona Diagnostic GmbH)) performed on nasopharyngeal swab and/or on autoptic samples. Demographics and clinical course of patients were depicted in Table S1. Patients age ranged between 27 and 92 years, the median age was 73 years. Most of the patients were men (*n* = 14; 70%; Table S1).

Autopsies were performed according to guidance for post-mortem collection and submission of specimens and biosafety practices^46^ to reduce the risk of transmission of infectious pathogens during and after the post-mortem examination. The study was approved by the local Clinical Research Ethics Committee (approval number: no 9/2020). Written informed consent was waived by the Ethics Commission due to public health outbreak investigation.

Specimens from lungs tissues were fixed in 10% neutral-buffered formalin, and routinely processed to paraffin blocks. Sections of tissues (4 μm) were stained with hematoxylin and eosin (H&E). For immunohistochemistry deparaffinized and rehydrated sections were used. Immunostaining was performed on BenchMark ULTRA system fully automated instrument (Roche) with antibody directed against anti-Coronavirus FIPV3-70 (Santa Cruz, sc-65653) or anti-dsRNA mAb (SCICONS J2).

### Transmission electron microscopy

Transmission electron microscopy (TEM) was performed on cultured cells and autopsy lung specimens using standard procedures. The ultrastructural analysis was performed on 4 out of 20 cases considered for this study, which resulted with positive SARS-CoV-2 PCR test on the lung tissue. Cultured cells and small pieces of tissues were fixed with 2.5% glutaraldehyde in 0.1 M cacodylate buffer, for 4 h at 4 °C. Post-fixation was performed with 1% OsO_4_. Samples were then dehydrated in graded ethanol and embedded in Epon resin, as previously described^47,48^. Ultrathin sections were stained with 2% uranyl acetate and observed under a transmission electron microscope JEOL JEM 2100 Plus (Japan Electron Optics Laboratory Co. Ltd. Tokyo, Japan). Images were captured digitally with a digital camera TVIPS (Tietz Video and Image Processing Systems GmbH. Gauting, Germany).

LD number and LD-mitochondria contacts were counted by analyzing at least 30 cells per each condition, at the electron microscope, assessed in blind by two authors.

### Confocal laser-scanning microscopy

Immunofluorescence, for double staining of viral infection and lipid droplets, was performed on SARS-CoV-2-infected Vero cells and lung samples from those cases which resulted with positive SARS-CoV-2 PCR test on the lung tissue (4 out of 20 cases).

To this aim infected cells fixed with 3% paraformaldehyde in PBS and immersed in 10 mM sodium citrate, pH 6.0, and microwaved for antigen retrieval were processed for confocal microscopy. Cryostat lung sections were fixed with 4% paraformaldehyde in PBS. Samples were incubated with mouse anti-dsRNA mAb (SCICONS J2), which is specific for dsRNA, overnight at 4 °C, and then incubated with Alexa Fluor 594 conjugated secondary antibody. For the detection of lipid droplets BODIPY 493/503 (2.5 um/ml) was applied. Samples were counterstained with DAPI (Invitrogen, Thermo Fisher Scientific).

The extent of coronavirus immunoreactivity and quantitative assessment of cells containing lipid droplets were assessed in blind by two authors, using at least five fields per sample (×63 objective). Data are the mean of three independent experiments, and each experimental point was run in duplicate. We conducted appropriate negative controls in parallel, using non infected samples and normal goat serum in place of the primary antibody.

Confocal fluorescence microscopy images were acquired on a Zeiss 900 LSM confocal equipped with an Airyscan2 detector, and processed using Zen software (Zeiss, Germany).

### Statistical analysis

Statistical significance between two groups under equivalent conditions was analyzed by two-sided, unpaired Student’s *t* test. *P* value < 0.05 was considered statistically significant. Data were represented as mean ± standard deviation (SD) of at three independent experiments.

## Supplementary information

Supplementary Material

Figure S1

Figure S2

Figure S3

Figure S4

Figure S5

Table S1

## Data Availability

All relevant data are within the manuscript and its Supporting Information files.
