# Supplementary material for: Evidences for lipid involvement in SARS-CoV-2 cytopathogenesis
Source: Cell Death Dis. 2021 Mar 12;12(3):263. doi: 10.1038/s41419-021-03527-9 (PMC7952828; doi:10.1038/s41419-021-03527-9)
Supplement: Supplementary file 1 — Supplementary Material [file 41419_2021_3527_MOESM1_ESM.doc]

**“Evidences for lipid involvement in SARS-CoV-2 cytopathogenesis”.**

**Supplementary Material**

**Figure S1. Negative staining electron microscopy micrographs of SARS-CoV-2 particles.**

Virions display a spherical (B) or slightly pleomorphic (A,C,D) shape. On the surface cone-like shaped spike are visible (arrows). Sometimes the envelope rupture allow nucleic material extruding from the surface (C).

Scale bars: 100 nm

**Figure S2. Cytophatic effects of SARS-CoV-2 and SARS-CoV-1 infection in Vero E6 cells.**

(A,B) SARS-CoV-2 infected Vero cells undergo progressive cytopathic effect from 24 h.p.i. to 48 h.p.i. becoming round and detaching from the culture flask. (C,D) SARS-CoV-1 infected cells displayed similar onset and extent of cytopathic effect. (E,F) Uninfected, control Vero cells.

**Figure S3. Light microscopy images of SARS-CoV-2 infected Vero E6 cells.**

(A) Control cells show the typical flattened shape. (B) SARS-CoV-2 infected cells at 24 hours post-infection display extensive roundish (arrows) and cell surface protrusions (arrowheads). (C,D) At 48 hours post-infection the cell morphology change further dramatically. Most cells appear rounded and swollen, with numerous cytoplasmatic vacuoles visible in some of them (arrows); as opposite other cells appear condensed (arrowheads).

Scale bars: A= 14 μm; B-D=7 μm

**Figure S4. Immunofluorescence dual-labeling detection of viral dsRNA and lipid droplets in lung tissue from COVID-19 patients.**

(A-C) Lung tissue from COVID-19 patients showing viral RNA (red) and lipids (green). Lipid droplets (green dots) are visible in the cytoplasm of numerous cells. (D) The image shows capillaries containing red blood cells (white arrows). The endothelial cells display absence of dsRNA signal.

dsRNA (red); lipids (green). Nuclei are stained blue (DAPI).

Scale bars: A= 14 μm; B-D=7 μm

**Figure S5. Quantification of lipid droplets and lipid droplets/mitochondria contacts in SARS-CoV-2-infected Vero cells.**

(A) The graph shows the mean number of lipid droplets contained in single cells at 24h and 48h post-infection. Data are mean + SD from at least three independent experiments, and each experiment included duplicate samples. Statistically significant difference is showed (*p<0.05).

(B) Number of cells presenting contacts between lipid droplets and mitochondria are shown.

**Table S1 Demographic and clinical features of COVID-19 patients**
