# Supplementary figures and images for: Evidences for lipid involvement in SARS-CoV-2 cytopathogenesis
Source: Cell Death Dis. 2021 Mar 12;12(3):263. doi: 10.1038/s41419-021-03527-9 (PMC7952828; doi:10.1038/s41419-021-03527-9)

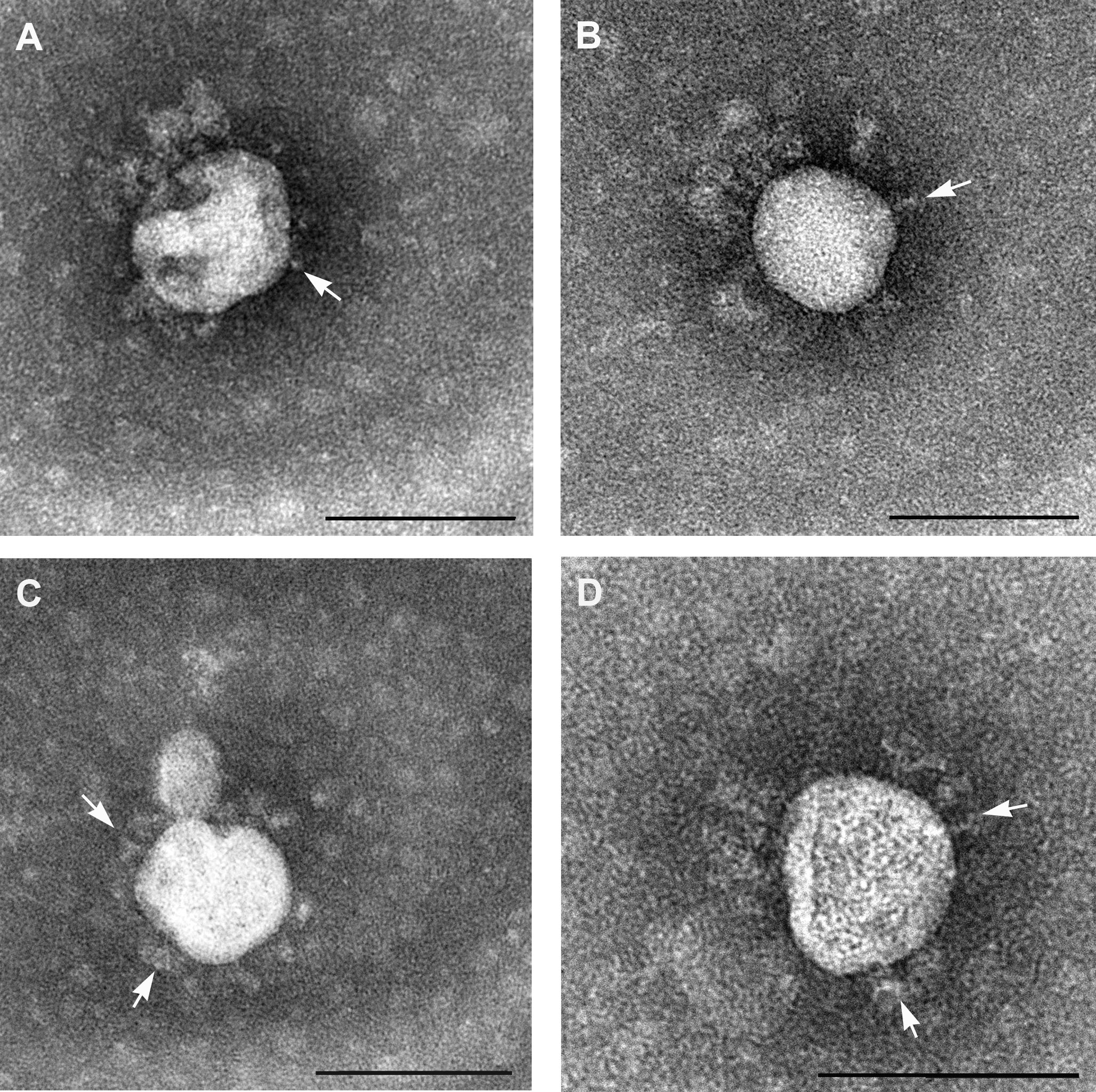

Supplement: Supplementary file 2 — Figure S1 [file 41419_2021_3527_MOESM2_ESM.tif]

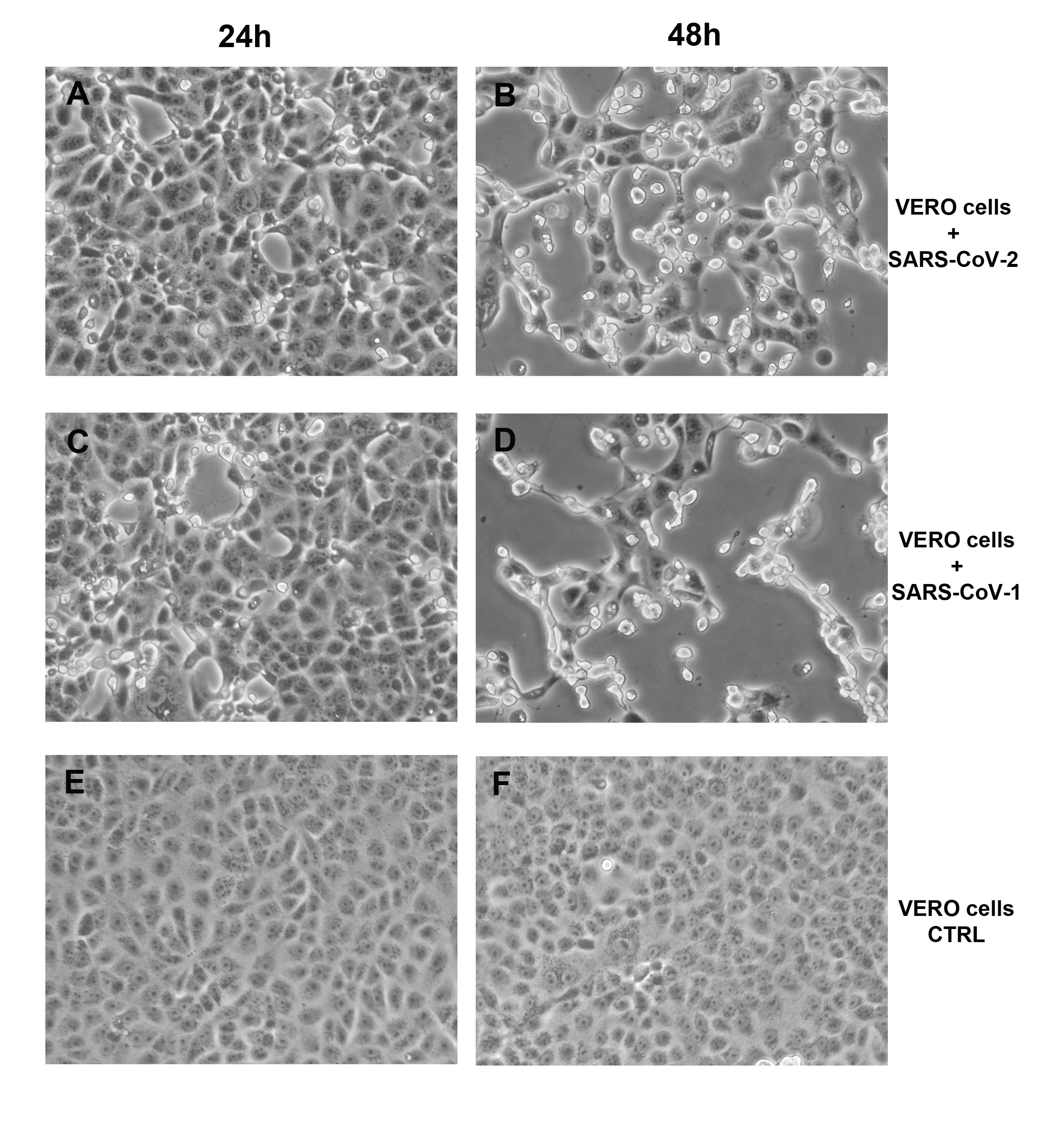

Supplement: Supplementary file 3 — Figure S2 [file 41419_2021_3527_MOESM3_ESM.tif]

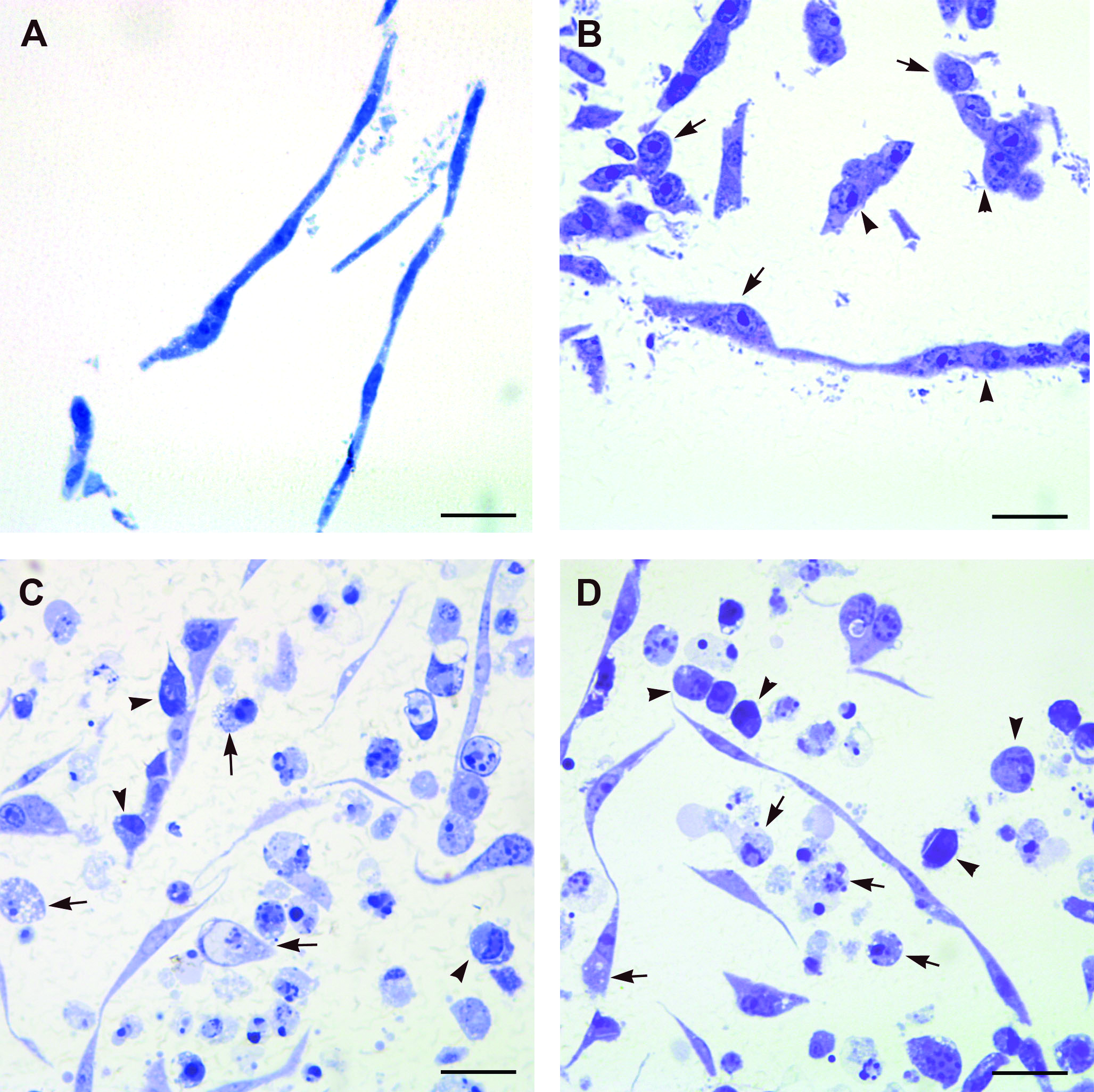

Supplement: Supplementary file 4 — Figure S3 [file 41419_2021_3527_MOESM4_ESM.tif]

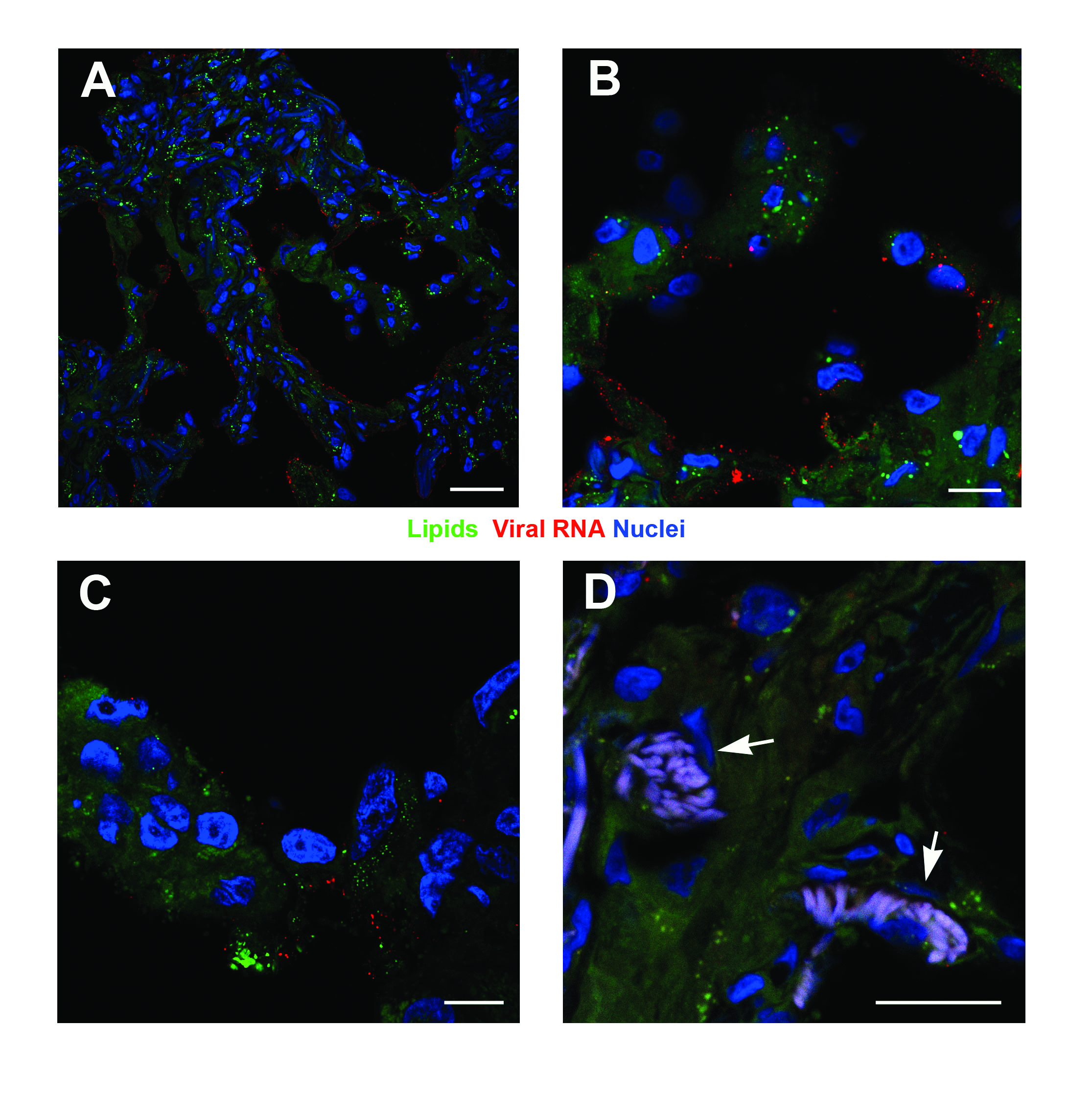

Supplement: Supplementary file 5 — Figure S4 [file 41419_2021_3527_MOESM5_ESM.tif]

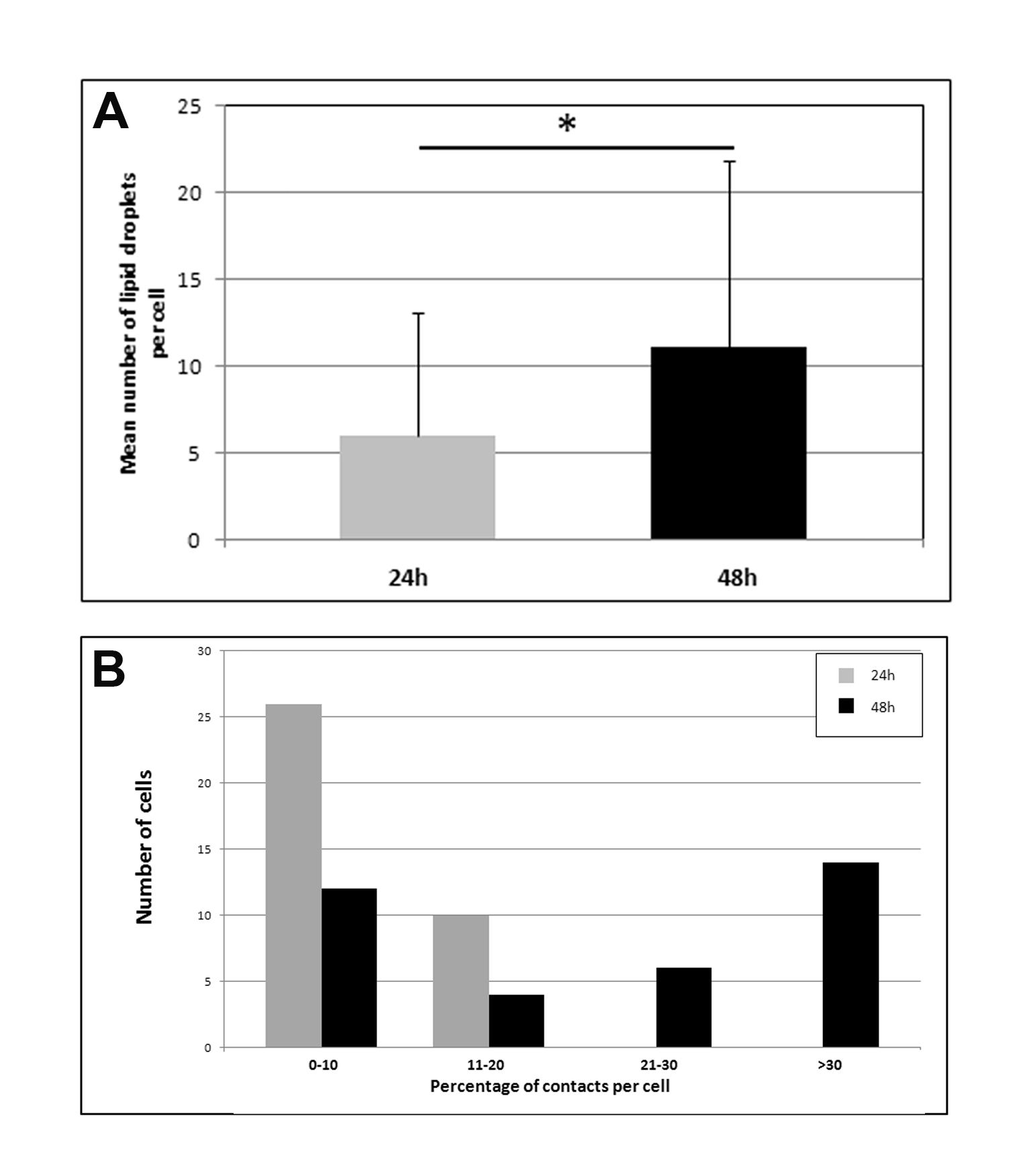

Supplement: Supplementary file 6 — Figure S5 [file 41419_2021_3527_MOESM6_ESM.tif]
