# Supplementary material for: Evidences for lipid involvement in SARS-CoV-2 cytopathogenesis
Source: Cell Death Dis. 2021 Mar 12;12(3):263. doi: 10.1038/s41419-021-03527-9 (PMC7952828; doi:10.1038/s41419-021-03527-9)
Supplement: Supplementary file 7 — Table S1 [file 41419_2021_3527_MOESM7_ESM.docx]

**Table S1 Demographic and clinical features of COVID-19 patients**

| **Patient Number** | **Gender** | **Age** | **Onset of syntoms**  **(days)** | **Comorbidities** | **Hospital stay (days)** | **Postmortem causes of death** | **Laboratory findings * (day before death)** | | | | |
| --- | --- | --- | --- | --- | --- | --- | --- | --- | --- | --- | --- |
|  |  |  |  |  |  |  | **Fibrinogen** | **D-Dimer** | **Lymphocyte** | **C-Recative protein** | **CPK** |
| **1** | M | 81 | Not known | Hypertension  Cardiomyopathy  Aortic aneurysm | 4 | Myocardial infarction. Diffuse alveolar damage (ARDS). Interstitial pneumonia | 634 | - | 0.330 | 21.8 | 83 |
| **2** | F | 69 | Not known | Schizophrenia  Staphylococcus | 28 | Cardiorespiratory failure | 112 | 1.24 | 1.95 | - | - |
| **3** | F | 82 | 3 | Atrial fibrillation. BPCO.Meningioma | 3 | Chronic pulmonary heart from obstructive pulmonary disease (COPD). Aorta stenosis. Fibrous pericarditis. Bilateral pleural effusion. Interstitial pneumonia | 853 | 595 | 0.490 | 25.2 | 99 |
| **4** | M | 92 | 7 | None | 1 | Cardiorespiratory failure | - | 480 | 1.27 | 0.51 | 161 |
| **5** | M | 54 | 4 | None | 16 | Interstitial pneumonia. Myocarditis | 722 | 3210 | 0.750 | 22.9 | 269 |
| **6** | M | 86 | Not known | Aortic aneurysm | 1 | Acute severe respiratory failure | - | - | 10.5 | 14.59 | - |
| **7** | M | 35 | 5 | None | 7 | CID. Interstitial pneumonia. Myocarditis | 634 | 1655 | 1.750 | 178.9 | 910 |
| **8** | F | 90 |  | Dementia. Ischemic heart disease | 33 | Cardiorespiratory failure | 150 | 2162 | 4.2 | 4.86 | 257 |
| **9** | M | 92 | 3 | Atrial fibrillation. Dementia.Osteomyelitis | 1 | Bilateral Interstitial Pneumonia. Broncopneumonia. Aortic aneurysm | 666 | 931 | 0.710 | 13.5 | 160 |
| **10** | F | 64 | 4 | Lobectomy for lung carcinoma | 1 | Respiratory failure for lung fibrosis | 599 | 491 | 0.770 | 1.77 | 275 |
| **11** | M | 58 | 3 | None | 1 | Bilateral interstitial pneumonia associated to pulmonary thrombosis. Myocarditis | 561 | 19717 | 1.420 | 12.5 | 71 |
| **12** | M | 64 | 28 | Myelodysplsia | 4 | Cardiorespiratory failure | >555 | 4610 | 1.78 | 37.31 | - |
| **13** | M | 82 | Not known | COPD  Aortic aneurysm | 4 | Cardiorespiratory failure | - | - | - | - | - |
| **14** | M | 76 | Not known | Malignancy | 30 | Cardiorespiratory failure | 194 | 1069 | 18.3 | 4.1 | 90 |
| **15** | M | 60 | 4 | Hypertension | 29 | Bilateral interstitial pneumonia  Heart failure | 666 | 4074 | 17.2 | 29 | 109 |
| **16** | F | 70 | Not known | Cardiopathy  Diabetes  Psychiatric pathology | 72 | Bilateral interstitial pneumonia  Pleural and pericardial effusion | 531 | 1519 | 9.2 | 4.44 | 231 |
| **17** | M | 76 | 3 | Malignancy  Resistant enterococcus | 33 | Cardiorespiratory failure | 503 | 4104 | 5.5 | 1.35 | 910 |
| **18** | F | 27 | 14 | HIV infection | 6 | Cryptococcosis | 280 | 4952 | 0.63 | 0.14 | 159 |
| **19** | M | 82 | Not known | Hypertension  Parkinson’s disease  Transient Ischemic Attacks | 14 | Bilateral interstitial pneumonia  Heart failure | 761 | 998 | 3.6 | 147.50 | - |
| **20** | M | 57 |  | Diabetes | 1 | Cardiorespiratory failure | - | - | - | - | - |

*Normal Values: Lymphoyte count x 10 ^3^ (1-3.5); D-Dimer ng/mL (0-500); Fibrinogen mg/dL (150-400); LDH U/L (200-400); PCR mg/dL (0.0 – 0.5); CPK U/L (52-336 M; 38-176 F)
